# Molecular identification of *Coranus* spp. in a pregnant patient: A case of misidentified Chagas disease vector in Spain

**DOI:** 10.1016/j.parepi.2025.e00426

**Published:** 2025-04-25

**Authors:** Jorge Ligero-López, María Dolores Bargues, Patricio Artigas, Giulia Colangeli, Fabiola Peiró-Codina, María Ducons-Márquez, Beatriz López-Alonso, Pilar Goñi, Antonio Beltrán-Rosel

**Affiliations:** a Clinical Microbiology Department, Hospital Clínico Universitario Lozano Blesa, Zaragoza, Spain; b Department of Microbiology, Pediatrics, Radiology and Public Health, Faculty of Medicine, Universidad de Zaragoza, Zaragoza, Spain; c Deparment of Parasitology, Faculty of Pharmacy, Universidad de Valencia, 46100 Burjassot, Valencia, Spain; d Centro de Investigación Biomédica en Red de Enfermedades Infecciosas (CIBERINFEC), Instituto de Salud Carlos III, 28029 Madrid, Spain; e Épila Health Center, Zaragoza, Spain; f Group of Water and Environmental Health, Institute of Environmental Sciences (IUCA), Spain

**Keywords:** Chagas disease, Triatominae, *Coranus* spp., Vector misidentification, *Trypanosoma cruzi*, Vector-borne diseases

## Abstract

Chagas disease is a significant public health concern in the Americas, transmitted primarily by vectors of the Triatominae subfamily. While Europe, particularly Spain, is free from endemic vectors, the potential for misidentification of non-hematophagous insects as Chagas vectors exists, leading to unnecessary alarm. We present the case of a 31-year-old pregnant Venezuelan woman residing in Spain, who sought medical attention after being bitten by an arthropod she identified as *Triatoma infestans*. The patient's awareness of Chagas disease in her country of origin heightened her concern about vertical transmission of *Trypanosoma cruzi* to her fetus. However, serological testing for *T. cruzi* antibodies was negative. The insect was initially misidentified as *T. infestans* but was later confirmed through molecular analysis to be *Coranus* spp., a non-hematophagous reduviid predator. The 18S rRNA gene sequence revealed a 99.37 % similarity to *Coranus* spp., ruling out any vectorial capacity for Chagas disease. This case underscores the importance of accurate arthropod identification, especially in non-endemic regions, to prevent misdiagnosis and unnecessary treatment. From a public health perspective, the introduction of a Chagas disease vector into Spain would represent a serious threat, necessitating prompt identification and containment measures. Our findings highlight the challenges posed by invasive species and the need for vigilance in regions where Chagas disease is not endemic. Proper identification of suspected vectors is crucial to ensure appropriate clinical and public health responses, preventing unwarranted anxiety and ensuring accurate disease surveillance.

## 1 Introduction

Chagas disease is transmitted by vectors belonging to the Reduviidae family, specifically the Triatominae subfamily. The principal genera implicated in transmission within endemic regions are *Rhodnius*, *Triatoma* and *Panstrongylus* (Martínez-Ibarra et al., 2008; Pérez-Molina and Molina, 2018)*)*. These genera contribute to the endemic status of the disease in 21 countries across the Americas, affecting approximately 6 million people and resulting in 30,000 new cases annually (PAHO, 2021; World Health Organization, 2024). The prevalence of *Trypanosoma cruzi* is highest in Bolivia, Argentina, Paraguay, Ecuador, El Salvador, and Guatemala (World Health Organization, 2015). Europe, particularly Spain, is currently free from the vectors responsible for Chagas disease; however, other non-hematophagous insect species can be mistaken for these vectors. We present a clinical case of an adult woman bitten by *Coranus* spp., a bug very similar in appearance to *Triatoma infestans*, which caused significant anxiety and concern in a pregnant patient.

## 2 Case presentation

We report the case of a 31-year-old Venezuelan woman residing in Spain for the past seven years. In May 2023, she visited the emergency department after experiencing an arthropod bite on her chest while at home. She captured the insect and, after conducting an internet search, identified it as *T. infestans*. The patient, who was 30 weeks pregnant, remained asymptomatic but was particularly concerned about the possibility of *Trypanosoma cruzi* transmission due to her awareness of Chagas disease in her country of origin. Her pregnancy heightened her concern regarding the potential for vertical transmission of *T. cruzi*. The patient reported no recent travel outside of Spain or contact with visitors from endemic areas. A blood sample obtained three days after the bite as part of gestational screening showed no detectable anti-*Trypanosoma cruzi* antibodies.

The emergency department ruled out urgent obstetric pathology and reassured the patient, explaining that Spain is not an endemic area for Chagas disease and does not have the necessary vector. A wait-and-see approach was adopted. The pregnancy proceeded normally, culminating in a cesarean section, and neonatal examination at birth revealed no abnormalities.

**Fig. 1:**
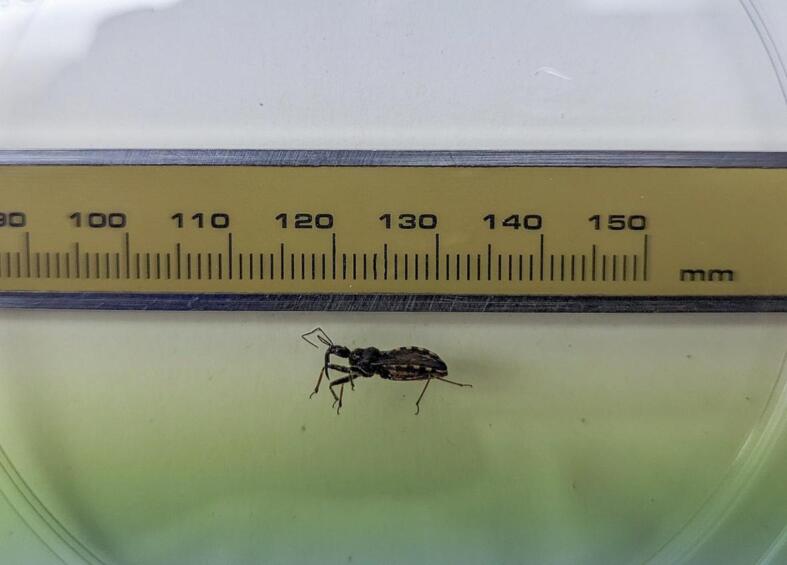
Approximate measurements of the arthropod.

**Fig. 2:**
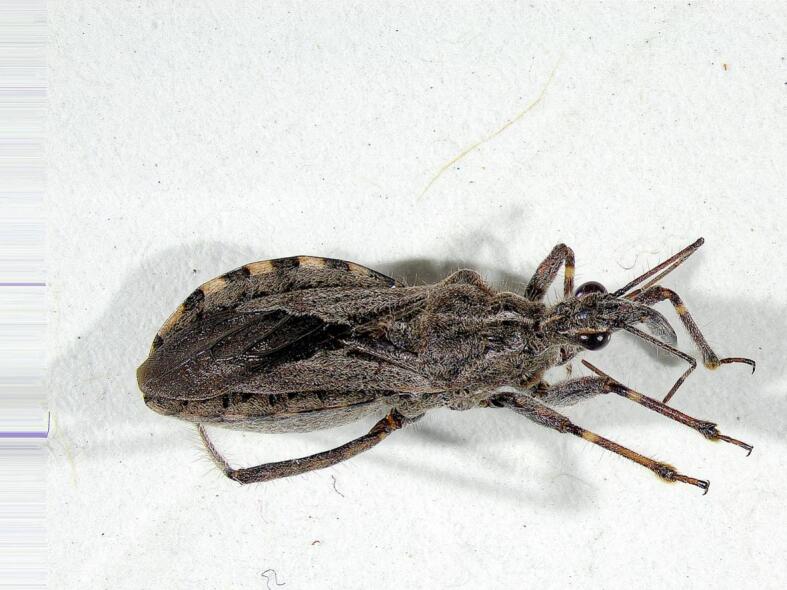
*Coranus* spp. Captured specimen.

The arthropod was sent to the Microbiology Service for identification. It was a bedbug approximately 10 mm in size, black with yellow and black two-toned edges (Fig. 1). An internet search revealed a strong resemblance to *T. infestans* (Fig. 2). Due to limited expertise, the specimen was sent to the Faculty of Pharmacy at the University of Valencia for further identification.

For DNA extraction, two legs from the specimen, fixed in 70 % ethanol, were used and processed individually. The complete sequence of the 18S rRNA gene was amplified using primers and PCR conditions as previously described (Mas-Coma and Bargues, 2009). Purified PCR products were resuspended in 50 μL of 10 mM TE buffer (pH 7.6). The final DNA concentration (in μg/mL) and absorbance at 260/280 nm were determined using an Eppendorf BioPhotometer (Hamburg, Germany). Sequencing was performed on both strands using the dideoxy chain-termination method, with the Taq dye-terminator chemistry kit on an Applied Biosystems 3730xl DNA Analyzer (Applied Biosystems, Foster City, CA, USA). Sequences were assembled and aligned using Clustal W with default parameters in MEGA X software. Homologies were determined using the BLASTN program from the National Center for Biotechnology Information website (http://www.ncbi.nlm.nih.gov/BLAST).

Comparative analysis was performed with 28 sequences of 18S, including 16 species of the main Chagas disease vectors, representatives of the four tribes of Triatomini, and 12 other reduviid predators from different genera, downloaded from GenBank and the haplotype collection of the Valencia Reference Center.

The sequence of the 18S rRNA gene obtained (1912 bp long, with 48.27 % GC content) showed 99.37 % similarity, with a BLAST coverage of 99 %, to the genus *Coranus* (GenBank Acc. No. OR907251), an assassin bug of the family Reduviidae, subfamily Harpactorinae. Comparative analysis of the sequences with other species of Chagas disease vectors and other predatory reduviids confirmed that the Zaragoza specimen was genetically much more distant from the genera *Triatoma*, *Rhodnius*, or *Panstrongylus* (p-distances = 0.036–0.038) than from the genus *Coranus* (p-distance = 0.010).

## 3 Discussion

**Fig. 3:**
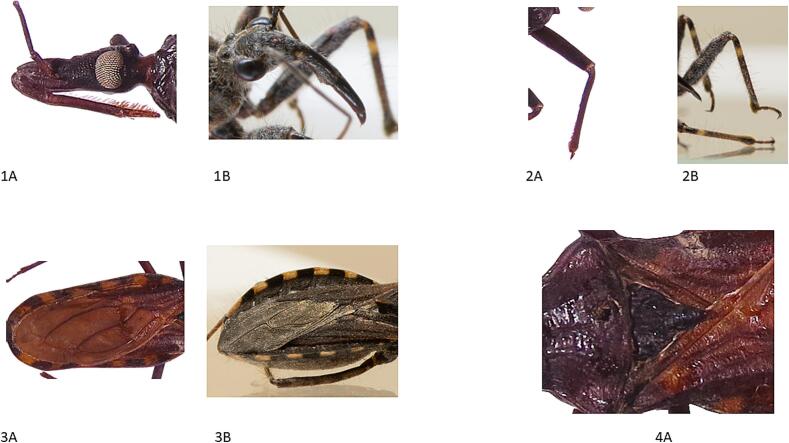
Morfological differences between *Coranus* spp. and *Triatoma infestans*. Panels 1 A and 1B illustrate differences in the head, eyes, and horn between the two species: 1 A – *T. infestans*, 1B – *Coranus* spp. Panels 2 A and 2B highlight the distinct hind leg morphology of *T. infestans* and *Coranus* spp.: 2 A – *T. infestans*, 2B – *Coranus* spp. Panels 3 A and 3B show variations in abdominal coloration between the two genera: 3 A – *T. infestans*, 3B – *Coranus* spp. Panel 4 A depicts the characteristic small, triangular scutellum of *T. infestans*.

**Fig. 4:**
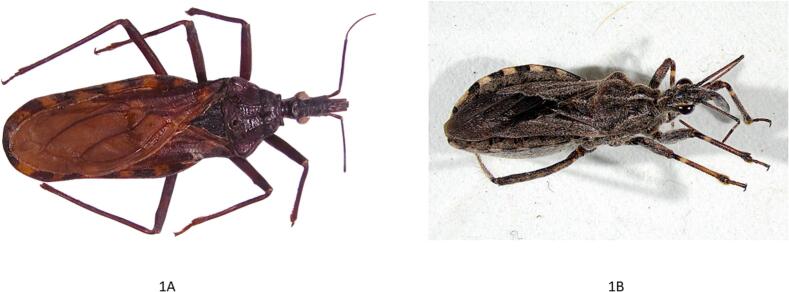
Complete bodies of both species, highlighting clear differences in the head, eyes, horns, coloration, and hind legs. 1 A – *Triatoma infestans*, 1B – *Coranus* spp. Original photos, courtesy of Patricio Artigas.

Species of the genus *Coranus* are not competent vectors for *T. cruzi*, and this information was conveyed to the patient. The genus *Coranus* is characterized by its primarily predatory habits and plays a significant role in regulating populations of other arthropods. In Aragón, Spain, six species of *Coranus* have been described: *Coranus griseus*, *C. kerzhneri*, *C. niger*, *C. pericarti*, *C. subapterus*, and *C. woodrofei* (Baena and Dos, 2022). In Barcelona they were also described (Goula et al., 2014). These species do not transmit known diseases and are not hematophagous; in this case, the bite likely occurred as a defensive response to feeling threatened. Some distinguishing characteristics between *Coranus* spp. and *T. infestans* are presented in Table 1**.** Additionally, the differences highlighted in the Table 1 can be seen in Fig. 3 and Fig. 4.

**Table 1:** Comparative Characteristics of *Coranus* spp. and *Triatoma infestans.*

Characteristics	*Coranus* spp.	*Triatoma infestans*
Size	9.5–14.2 mm	Up to 35 mm
Coloration	Variable depending on species, with dark patterns on the ventral abdomen or light lateral bands.	Dark brown with yellow or red margins on the belly.
Head	Shorter and wider.	Elongated, joined to the thorax by a narrow neck.
Eyes	Prominent, lateral, and non-compound.	Large, prominent compound eyes with vestigial ocelli always present.
Horn	Short, strong, and curved backward.	Long, slender, and straight.
Pronotum	Anterior lobe with two or three longitudinal grooves, irregularly parallel to the median groove, which is well marked.	The pronotum has grooves that distinguish an anterior and posterior lobe, the former being much shorter.
Scutellum	Rectangular, curved, or apophysed depending on the species. Some species may have a long hooked projection.	Small and triangular.
Front legs	Thorny, with pseudopinches for holding prey.	Not thorny, without any special modifications for prey trapping.
Elytra	Hemielytra; they completely cover the abdomen in macropterous species; some species may be brachypterous.	Hemielytra; always macropterous, covering the abdomen.
Eating habits	Insect predator	Hematophagous.
Medical importance	No known medical significance	*Trypanosoma cruzi* vector, the cause of Chagas disease
Distribution	Europe	South America

The correct identification of the arthropod was particularly important. Had the arthropod been unequivocally identified as a vector of *T. cruzi*, there would have been a possibility of gestational primoinfection and vertical transmission to the fetus, necessitating antiparasitic treatment. From a public health perspective, the introduction of a species with vectorial capacity for Chagas disease into Spain—a country with a significant population affected by Chagas disease—would constitute a significant health emergency.

The invasive capacity of various arthropod groups is well documented, with notable examples including *Aedes albopictus* (Artigas et al., 2021, De-Elías-Escribano et al., 2025; Oliveira et al., 2021) and *Haemaphysalis longicornis* (Rochlin et al., 2023). In Spain, migratory birds and passively transported ticks have been implicated in the expansion of the Crimean-Congo hemorrhagic fever virus (Palomar et al., 2013). Although triatomine mobility is generally limited, they can be passively transported via infested goods, animals, luggage, and air transport routes, expanding their territory (Pinto Dias, 2013). This has been documented in three species, all of which are competent vectors for *T. cruzi*: *Triatoma rubrofasciata*, widely distributed and recorded in the USA, Central and South America, Africa, the Middle East, and the Azores, believed to have dispersed along shipping routes between the 16th and 19th centuries due to its association with domestic rats (Dujardin et al., 2015); *Rhodnius prolixus*, which was likely transported from Venezuela to Mexico and Central America through maritime trade and possibly bird migration (Hashimoto and Schofield, 2012) and *T. infestans* which is speculated to have reached Mexico from South America via a shipment of seeds in a twenty-foot equivalent unit (TEU) shipping container (Martínez-Hernández et al., 2022).

Outside the Americas, information on the climatic suitability for these vectors to establish and spread is lacking. In Europe, the presence of kissing bugs transmitting Chagas disease has not been recorded as endemic. However, the environmental conditions of southern Europe could be suitable for *Triatoma sordida* and *T. infestans*. In addition to climatic conditions, many triatomine species have specific microhabitat preferences and host spectra, making only a few species capable of adapting to other environments (Eberhard et al., 2020).

Correct identification of arthropods in endemic areas is clinically essential for offering early treatment when necessary and epidemiologically essential for mapping and tracking their expansion. Misidentification of non-Chagas-disease-vector bugs with vinchucas is common, as seen in Chile with *Leptoglossus occidentalis*, an invasive species mistakenly identified, causing social alarm (Faúndez et al., 2020). In the United States, scientists from Texas A&M University have created a comprehensive website highlighting the main differences between vinchucas and other bugs, allowing the public to submit specimens for analysis using molecular biology (Texas A&M University, n.d.). Additionally, public education and community engagement regarding the vectors that transmit Chagas disease have proven to be key strategies in controlling the disease in South and Central America (Abad-Franch et al., 2011), with promising results in Texas (Curtis-Robles et al., 2015). Similarly, educating healthcare professionals about Chagas disease enhances their knowledge and awareness, making them more vigilant (Stigler Granados et al., 2020).

In non-endemic countries, accurate identification is crucial for two main reasons. First, if the arthropod is confirmed as an invasive species with vector potential, appropriate diagnostic and therapeutic measures must be implemented. Furthermore, bed bug bites are rarely reported in medical settings, and microbiology services often have limited capacity to identify these arthropods, posing an additional challenge. In this regard, collaboration with expert entomologists—even from institutions outside Spain—or utilizing citizen science platforms such as the Texas A&M University website (Texas A&M University, n.d.) or iNaturalist (iNaturalist, 2024) can be valuable for clarifying uncertainties in triatomine identification. In Spain, the misidentification of local bedbug species with low medical significance as *T. infestans* appears to be a relatively common occurrence (personal communication). From a public health perspective, the presence of a vector species would require rigorous monitoring and the implementation of appropriate containment measures.

## Availability of data and materials

No datasets were generated or analyzed during the current study.

## CRediT authorship contribution statement

**Jorge Ligero-López:** Writing – review & editing, Writing – original draft, Methodology, Conceptualization. **María Dolores-Bargues:** Writing – review & editing, Writing – original draft, Methodology, Conceptualization. **Patricio Artigas:** Visualization, Methodology, Investigation. **Giulia Colangeli:** Visualization, Methodology, Investigation. **Fabiola Peiró-Codina:** Visualization, Methodology, Investigation. **María Ducons-Márquez:** Visualization, Methodology, Investigation. **Beatriz López-Alonso:** Visualization, Methodology, Investigation. **Pilar Goñi:** Visualization, Methodology, Investigation. **Antonio Beltrán-Rosel:** Writing – review & editing, Writing – original draft, Methodology, Conceptualization.

## Consent for publication

Not applicable.

## Ethics approval and consent to participate

Not applicable.

## Funding

This research was funded by: CIBER de Enfermedades Infecciosas (CB21/13/00056), 10.13039/501100004587ISCIII, 10.13039/501100004837Ministry of Science and Innovation, and European Union – NextGenerationEU, Madrid; and the PROMETEO Program, Programa de Ayudas para Grupos de Investigación de Excelencia (2021/004), 10.13039/501100003359Generalitat Valenciana, Valencia, Spain.

## Declaration of competing interest

The authors declare no competing interests.
